# 1-(3-Meth­oxy­phen­yl)-4,5-dimethyl-2-phenyl-1*H*-imidazole

**DOI:** 10.1107/S1600536813016966

**Published:** 2013-06-26

**Authors:** S. Rizwana Begum, R. Hema, N. Srinivasan, A. G. Anitha

**Affiliations:** a Department of Physics, Seethalakshmi Ramaswami College (Autonomous), Tiruchirappalli 620 002, India; b Department of Chemistry, S.K.P. Engineering College, Thiruvanamalai 606 611, India

## Abstract

In the title compound, C_18_H_18_N_2_O, the imidazole ring makes dihedral angles of 68.26 (7) and 22.45 (9)° with the meth­oxy­phenyl and phenyl rings, respectively. The dihedral angle between the meth­oxy­phenyl and phenyl ring is 71.86 (7)°. In the crystal, weak inter­molecular C—H⋯O and C—H⋯N hydrogen bonds link the mol­ecules into columns propagated in [101].

## Related literature

For related structures, see: Gayathri *et al.* (2010 ▶); Rosepriya *et al.* (2011 ▶). For graph-set motifs, see: Bernstein *et al.* (1995 ▶).
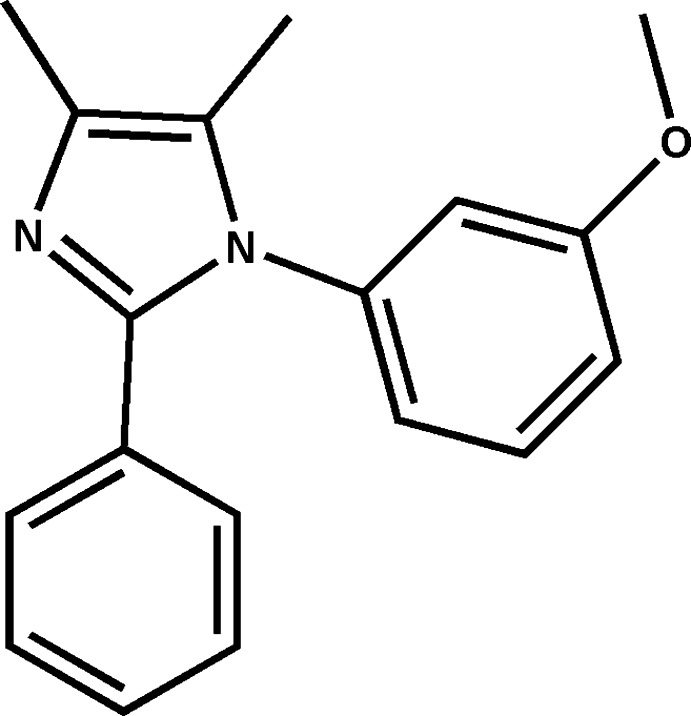


## Experimental

### 

#### Crystal data

C_18_H_18_N_2_O
*M* *_r_* = 278.34
Triclinic, 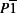

*a* = 8.0199 (1) Å
*b* = 9.4807 (1) Å
*c* = 10.4971 (2) Å
α = 108.339 (1)°
β = 94.910 (1)°
γ = 90.535 (1)°
*V* = 754.27 (2) Å^3^
*Z* = 2
Mo *K*α radiation
μ = 0.08 mm^−1^
*T* = 293 K
0.35 × 0.30 × 0.30 mm

#### Data collection

Bruker Kappa APEXII CCD diffractometer
Absorption correction: multi-scan (*SADABS*; Bruker, 2004 ▶) *T*_min_ = 0.974, *T*_max_ = 0.977
14252 measured reflections
2644 independent reflections
2159 reflections with *I* > 2σ(*I*)
*R*_int_ = 0.021

#### Refinement

*R*[*F* ^2^ > 2σ(*F* ^2^)] = 0.046
*wR*(*F* ^2^) = 0.148
*S* = 1.04
2644 reflections
191 parameters
H-atom parameters constrained
Δρ_max_ = 0.33 e Å^−3^
Δρ_min_ = −0.22 e Å^−3^

### 

Data collection: *APEX2* (Bruker, 2004 ▶); cell refinement: *APEX2* and *SAINT* (Bruker, 2004 ▶); data reduction: *SAINT* and *XPREP*; program(s) used to solve structure: *SIR92* (Altomare *et al.*, 1994 ▶); program(s) used to refine structure: *SHELXL97* (Sheldrick, 2008 ▶); molecular graphics: *ORTEP-3* (Farrugia, 2012 ▶); software used to prepare material for publication: *SHELXL97*.

## Supplementary Material

Crystal structure: contains datablock(s) I, global. DOI: 10.1107/S1600536813016966/cv5418sup1.cif

Structure factors: contains datablock(s) I. DOI: 10.1107/S1600536813016966/cv5418Isup2.hkl

Supplementary material file. DOI: 10.1107/S1600536813016966/cv5418Isup3.cml

Additional supplementary materials:  crystallographic information; 3D view; checkCIF report

## Figures and Tables

**Table 1:** Hydrogen-bond geometry (Å, °)

*D*—H⋯*A*	*D*—H	H⋯*A*	*D*⋯*A*	*D*—H⋯*A*
C5—H5*A*⋯O1^i^	0.96	2.57	3.316 (3)	135
C7—H7⋯N2^ii^	0.93	2.58	3.493 (2)	168

Symmetry codes: (i) 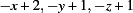; (ii) 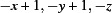.

**Fig. 1.**
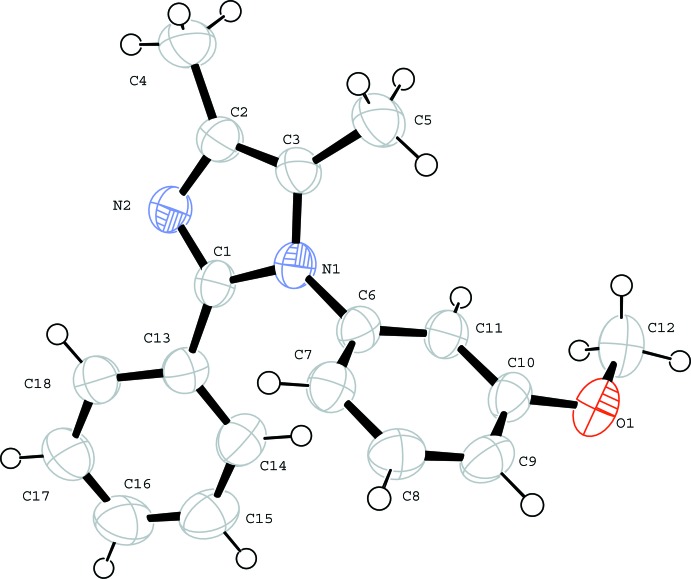
The molecular structure of the title compound,showing the atom-labelling scheme. Displacement ellipsoids are drawn at the 50% probability level. H atoms are represented by circles of arbitrary radii.

## supplementary crystallographic information

### Comment

In a continuation of structural studies of 4,5-dimethyl-1*H*-imidazole derivatives (Gayathri *et al.*, 2010; Rosepriya *et al.*, 2011), herewith we present the title compound, (I).

In (I) (Fig. 1), the imidazole ring is essentially planar [maximum deviation of 0.0036 (11) Å for N2 and -0.0036 (11) Å N1]. The imidazole ring makes dihedral angle of 68.26 (7)° and 22.45 (9)° with the methoxyphenyl (C6–C11) and phenyl (C13–C18) rings, respectively. The dihedral angle between the methoxyphenyl and phenyl rings is 71.86 (7)°.

The crystal structure is stabilized by weak C—H···O and C—H···N intermolecular interactions (Table 1). The C—H···O interactions link pairs of molecules across centres of inversion to give the ring motif *R*(16) (Bernstein *et al.*, 1995). Atom C7 acts as a donor for a weak intermolecular C—H···N interaction *via* H7 with the nitrogen atom in the imidazole moiety, thus forming extended chains with a graph set motif C(6) (Bernstein *et al.*, 1995).

### Experimental

To pure butane-2,3-dione (1.48 g, 15 mmol) in ethanol (10 ml), *m*-methoxy aniline (1.5 g, 15 mmol), ammonium acetate (1.15 g, 15 mmol) and benzaldehyde (1.5 g, 15 mmol) was added about 1 h by maintaining the temperature at 333 K. The reaction mixture was refluxed for 7 days and extracted with dichloromethane. The solid separated was purified by column chromatography using hexane: ethyl acetate as the eluent. Yield: 1.91 g (46%).

### Refinement

The methyl H atoms were constrained to an ideal geometry (C—H = 0.96 Å) with *U*_iso_(H) = 1.5*U*_eq_(C), but were allowed to rotate freely about the C—C bonds. All remaining H atoms were placed in geometrically idealized positions (C—H = 0.95–1.00 Å) and constrained to ride on their parent atoms with *U*_iso_(H) = 1.2*U*_eq_(C).

### Crystal data

**Table:**

C_18_H_18_N_2_O	*Z* = 2
*M**_r_* = 278.34	*F*(000) = 296
Triclinic, *P*1	*D*_x_ = 1.226 Mg m^−^^3^
Hall symbol: -P 1	Mo *K*α radiation, λ = 0.71073 Å
*a* = 8.0199 (1) Å	Cell parameters from 2644 reflections
*b* = 9.4807 (1) Å	θ = 2.3–30.6°
*c* = 10.4971 (2) Å	µ = 0.08 mm^−^^1^
α = 108.339 (1)°	*T* = 293 K
β = 94.910 (1)°	Block, colourless
γ = 90.535 (1)°	0.35 × 0.30 × 0.30 mm
*V* = 754.27 (2) Å^3^	

### Data collection

**Table:**

Bruker Kappa APEXII CCD diffractometer	2644 independent reflections
Radiation source: fine-focus sealed tube	2159 reflections with *I* > 2σ(*I*)
Graphite monochromator	*R*_int_ = 0.021
ω and φ scan	θ_max_ = 25.0°, θ_min_ = 2.1°
Absorption correction: multi-scan (*SADABS*; Bruker, 2004)	*h* = −9→9
*T*_min_ = 0.974, *T*_max_ = 0.977	*k* = −11→11
14252 measured reflections	*l* = −12→12

### Refinement

**Table:**

Refinement on *F*^2^	Secondary atom site location: difference Fourier map
Least-squares matrix: full	Hydrogen site location: inferred from neighbouring sites
*R*[*F*^2^ > 2σ(*F*^2^)] = 0.046	H-atom parameters constrained
*wR*(*F*^2^) = 0.148	*w* = 1/[σ^2^(*F*_o_^2^) + (0.0747*P*)^2^ + 0.2805*P*] where *P* = (*F*_o_^2^ + 2*F*_c_^2^)/3
*S* = 1.04	(Δ/σ)_max_ < 0.001
2644 reflections	Δρ_max_ = 0.33 e Å^−^^3^
191 parameters	Δρ_min_ = −0.22 e Å^−^^3^
0 restraints	Extinction correction: *SHELXL97* (Sheldrick, 2008), Fc^*^=kFc[1+0.001xFc^2^λ^3^/sin(2θ)]^-1/4^
Primary atom site location: structure-invariant direct methods	Extinction coefficient: 0.028 (7)

### Special details

**Table:**

Geometry. All e.s.d.'s (except the e.s.d. in the dihedral angle between two l.s. planes) are estimated using the full covariance matrix. The cell e.s.d.'s are taken into account individually in the estimation of e.s.d.'s in distances, angles and torsion angles; correlations between e.s.d.'s in cell parameters are only used when they are defined by crystal symmetry. An approximate (isotropic) treatment of cell e.s.d.'s is used for estimating e.s.d.'s involving l.s. planes.
Refinement. Refinement of *F*^2^ against ALL reflections. The weighted *R*-factor *wR* and goodness of fit *S* are based on *F*^2^, conventional *R*-factors *R* are based on *F*, with *F* set to zero for negative *F*^2^. The threshold expression of *F*^2^ > σ(*F*^2^) is used only for calculating *R*-factors(gt) *etc*. and is not relevant to the choice of reflections for refinement. *R*-factors based on *F*^2^ are statistically about twice as large as those based on *F*, and *R*- factors based on ALL data will be even larger.

### Fractional atomic coordinates and isotropic or equivalent isotropic displacement parameters (Å^2^)

**Table:**

	*x*	*y*	*z*	*U*_iso_*/*U*_eq_	
C1	0.7362 (2)	0.4690 (2)	−0.02515 (17)	0.0442 (4)	
C2	0.7291 (3)	0.7075 (2)	0.05116 (19)	0.0521 (5)	
C3	0.7842 (3)	0.6639 (2)	0.15818 (19)	0.0513 (5)	
C4	0.6975 (4)	0.8605 (2)	0.0466 (3)	0.0792 (7)	
H4A	0.6594	0.8561	−0.0438	0.119*	
H4B	0.7993	0.9205	0.0751	0.119*	
H4C	0.6135	0.9036	0.1057	0.119*	
C5	0.8313 (3)	0.7516 (3)	0.3018 (2)	0.0713 (7)	
H5A	0.8654	0.6856	0.3514	0.107*	
H5B	0.7368	0.8053	0.3393	0.107*	
H5C	0.9222	0.8206	0.3072	0.107*	
C6	0.8135 (2)	0.4174 (2)	0.19308 (17)	0.0440 (4)	
C7	0.6791 (2)	0.3354 (2)	0.21018 (19)	0.0500 (5)	
H7	0.5731	0.3408	0.1689	0.060*	
C8	0.7059 (3)	0.2451 (2)	0.2900 (2)	0.0569 (5)	
H8	0.6170	0.1879	0.3015	0.068*	
C9	0.8613 (3)	0.2385 (2)	0.3528 (2)	0.0557 (5)	
H9	0.8773	0.1770	0.4061	0.067*	
C10	0.9949 (2)	0.3234 (2)	0.33671 (17)	0.0483 (5)	
C11	0.9717 (2)	0.4125 (2)	0.25552 (17)	0.0466 (5)	
H11	1.0610	0.4685	0.2429	0.056*	
C12	1.2812 (3)	0.4015 (3)	0.3979 (2)	0.0701 (6)	
H12A	1.3781	0.3800	0.4479	0.105*	
H12B	1.2553	0.5040	0.4365	0.105*	
H12C	1.3037	0.3830	0.3058	0.105*	
C13	0.7294 (2)	0.3179 (2)	−0.12117 (18)	0.0482 (5)	
C14	0.8223 (3)	0.2026 (2)	−0.1019 (2)	0.0621 (6)	
H14	0.8895	0.2175	−0.0217	0.075*	
C15	0.8159 (3)	0.0654 (3)	−0.2010 (3)	0.0743 (7)	
H15	0.8778	−0.0114	−0.1864	0.089*	
C16	0.7189 (4)	0.0416 (3)	−0.3206 (3)	0.0770 (7)	
H16	0.7157	−0.0505	−0.3872	0.092*	
C17	0.6270 (3)	0.1548 (3)	−0.3408 (2)	0.0765 (7)	
H17	0.5617	0.1395	−0.4219	0.092*	
C18	0.6304 (3)	0.2911 (2)	−0.2422 (2)	0.0611 (6)	
H18	0.5654	0.3663	−0.2568	0.073*	
N1	0.78868 (18)	0.51065 (17)	0.10977 (14)	0.0450 (4)	
N2	0.70075 (19)	0.58661 (17)	−0.06211 (15)	0.0489 (4)	
O1	1.14352 (18)	0.30952 (17)	0.40312 (15)	0.0643 (4)	

### Atomic displacement parameters (Å^2^)

**Table:**

	*U*^11^	*U*^22^	*U*^33^	*U*^12^	*U*^13^	*U*^23^
C1	0.0460 (9)	0.0516 (11)	0.0371 (9)	0.0035 (7)	0.0034 (7)	0.0172 (8)
C2	0.0646 (12)	0.0465 (11)	0.0461 (11)	−0.0015 (8)	−0.0005 (8)	0.0172 (9)
C3	0.0627 (12)	0.0481 (11)	0.0416 (10)	−0.0036 (8)	−0.0016 (8)	0.0140 (8)
C4	0.119 (2)	0.0510 (13)	0.0667 (15)	−0.0024 (13)	−0.0097 (14)	0.0222 (11)
C5	0.1036 (18)	0.0594 (13)	0.0453 (12)	−0.0065 (12)	−0.0068 (11)	0.0125 (10)
C6	0.0527 (10)	0.0467 (10)	0.0339 (9)	0.0052 (8)	0.0062 (7)	0.0141 (8)
C7	0.0503 (10)	0.0528 (11)	0.0474 (10)	0.0035 (8)	0.0058 (8)	0.0163 (9)
C8	0.0627 (12)	0.0527 (12)	0.0594 (12)	−0.0002 (9)	0.0132 (9)	0.0217 (10)
C9	0.0733 (13)	0.0490 (11)	0.0520 (11)	0.0099 (9)	0.0110 (9)	0.0246 (9)
C10	0.0579 (11)	0.0516 (11)	0.0357 (9)	0.0134 (8)	0.0049 (8)	0.0136 (8)
C11	0.0499 (10)	0.0529 (11)	0.0395 (9)	0.0028 (8)	0.0061 (7)	0.0175 (8)
C12	0.0587 (13)	0.0918 (17)	0.0619 (14)	0.0079 (11)	−0.0043 (10)	0.0295 (13)
C13	0.0544 (10)	0.0507 (11)	0.0417 (10)	0.0035 (8)	0.0101 (8)	0.0162 (9)
C14	0.0744 (14)	0.0645 (14)	0.0477 (11)	0.0183 (11)	0.0104 (10)	0.0166 (10)
C15	0.0971 (18)	0.0581 (14)	0.0686 (15)	0.0237 (12)	0.0217 (13)	0.0173 (12)
C16	0.1025 (19)	0.0565 (14)	0.0621 (15)	0.0029 (12)	0.0126 (13)	0.0031 (11)
C17	0.0965 (18)	0.0652 (15)	0.0556 (14)	−0.0038 (13)	−0.0093 (12)	0.0061 (11)
C18	0.0733 (14)	0.0555 (12)	0.0511 (12)	0.0024 (10)	−0.0042 (10)	0.0147 (10)
N1	0.0508 (9)	0.0488 (9)	0.0371 (8)	0.0020 (6)	0.0013 (6)	0.0165 (7)
N2	0.0566 (9)	0.0512 (9)	0.0410 (8)	0.0011 (7)	0.0000 (7)	0.0189 (7)
O1	0.0634 (9)	0.0770 (10)	0.0610 (9)	0.0117 (7)	−0.0025 (7)	0.0360 (8)

### Geometric parameters (Å, º)

**Table:**

C1—N2	1.317 (2)	C9—C10	1.386 (3)
C1—N1	1.372 (2)	C9—H9	0.9300
C1—C13	1.467 (3)	C10—O1	1.358 (2)
C2—C3	1.356 (3)	C10—C11	1.380 (3)
C2—N2	1.368 (2)	C11—H11	0.9300
C2—C4	1.490 (3)	C12—O1	1.416 (3)
C3—N1	1.382 (2)	C12—H12A	0.9600
C3—C5	1.488 (3)	C12—H12B	0.9600
C4—H4A	0.9600	C12—H12C	0.9600
C4—H4B	0.9600	C13—C14	1.386 (3)
C4—H4C	0.9600	C13—C18	1.389 (3)
C5—H5A	0.9600	C14—C15	1.383 (3)
C5—H5B	0.9600	C14—H14	0.9300
C5—H5C	0.9600	C15—C16	1.373 (4)
C6—C7	1.380 (3)	C15—H15	0.9300
C6—C11	1.385 (2)	C16—C17	1.368 (4)
C6—N1	1.431 (2)	C16—H16	0.9300
C7—C8	1.380 (3)	C17—C18	1.377 (3)
C7—H7	0.9300	C17—H17	0.9300
C8—C9	1.371 (3)	C18—H18	0.9300
C8—H8	0.9300		
			
N2—C1—N1	110.51 (16)	O1—C10—C9	115.68 (17)
N2—C1—C13	122.63 (16)	C11—C10—C9	119.90 (17)
N1—C1—C13	126.73 (16)	C10—C11—C6	119.00 (17)
C3—C2—N2	110.20 (17)	C10—C11—H11	120.5
C3—C2—C4	128.78 (19)	C6—C11—H11	120.5
N2—C2—C4	121.02 (17)	O1—C12—H12A	109.5
C2—C3—N1	105.96 (16)	O1—C12—H12B	109.5
C2—C3—C5	130.95 (19)	H12A—C12—H12B	109.5
N1—C3—C5	123.08 (17)	O1—C12—H12C	109.5
C2—C4—H4A	109.5	H12A—C12—H12C	109.5
C2—C4—H4B	109.5	H12B—C12—H12C	109.5
H4A—C4—H4B	109.5	C14—C13—C18	117.98 (19)
C2—C4—H4C	109.5	C14—C13—C1	124.16 (18)
H4A—C4—H4C	109.5	C18—C13—C1	117.77 (17)
H4B—C4—H4C	109.5	C15—C14—C13	120.5 (2)
C3—C5—H5A	109.5	C15—C14—H14	119.7
C3—C5—H5B	109.5	C13—C14—H14	119.7
H5A—C5—H5B	109.5	C16—C15—C14	120.6 (2)
C3—C5—H5C	109.5	C16—C15—H15	119.7
H5A—C5—H5C	109.5	C14—C15—H15	119.7
H5B—C5—H5C	109.5	C17—C16—C15	119.3 (2)
C7—C6—C11	121.60 (17)	C17—C16—H16	120.3
C7—C6—N1	119.22 (16)	C15—C16—H16	120.3
C11—C6—N1	119.18 (16)	C16—C17—C18	120.6 (2)
C6—C7—C8	118.36 (18)	C16—C17—H17	119.7
C6—C7—H7	120.8	C18—C17—H17	119.7
C8—C7—H7	120.8	C17—C18—C13	120.9 (2)
C9—C8—C7	121.02 (19)	C17—C18—H18	119.5
C9—C8—H8	119.5	C13—C18—H18	119.5
C7—C8—H8	119.5	C1—N1—C3	106.80 (15)
C8—C9—C10	120.11 (18)	C1—N1—C6	127.92 (15)
C8—C9—H9	119.9	C3—N1—C6	124.33 (15)
C10—C9—H9	119.9	C1—N2—C2	106.53 (15)
O1—C10—C11	124.41 (18)	C10—O1—C12	117.98 (16)
			
N2—C2—C3—N1	−0.6 (2)	C15—C16—C17—C18	−0.4 (4)
C4—C2—C3—N1	178.6 (2)	C16—C17—C18—C13	1.3 (4)
N2—C2—C3—C5	−179.7 (2)	C14—C13—C18—C17	−1.1 (3)
C4—C2—C3—C5	−0.5 (4)	C1—C13—C18—C17	175.5 (2)
C11—C6—C7—C8	−1.0 (3)	N2—C1—N1—C3	0.2 (2)
N1—C6—C7—C8	179.48 (16)	C13—C1—N1—C3	176.15 (17)
C6—C7—C8—C9	1.0 (3)	N2—C1—N1—C6	169.31 (16)
C7—C8—C9—C10	0.1 (3)	C13—C1—N1—C6	−14.8 (3)
C8—C9—C10—O1	179.81 (17)	C2—C3—N1—C1	0.2 (2)
C8—C9—C10—C11	−1.2 (3)	C5—C3—N1—C1	179.5 (2)
O1—C10—C11—C6	−179.95 (16)	C2—C3—N1—C6	−169.35 (17)
C9—C10—C11—C6	1.1 (3)	C5—C3—N1—C6	9.9 (3)
C7—C6—C11—C10	0.0 (3)	C7—C6—N1—C1	−61.6 (2)
N1—C6—C11—C10	179.45 (15)	C11—C6—N1—C1	118.9 (2)
N2—C1—C13—C14	153.96 (19)	C7—C6—N1—C3	105.7 (2)
N1—C1—C13—C14	−21.5 (3)	C11—C6—N1—C3	−73.8 (2)
N2—C1—C13—C18	−22.4 (3)	N1—C1—N2—C2	−0.6 (2)
N1—C1—C13—C18	162.10 (19)	C13—C1—N2—C2	−176.70 (16)
C18—C13—C14—C15	0.1 (3)	C3—C2—N2—C1	0.7 (2)
C1—C13—C14—C15	−176.29 (19)	C4—C2—N2—C1	−178.6 (2)
C13—C14—C15—C16	0.7 (4)	C11—C10—O1—C12	5.4 (3)
C14—C15—C16—C17	−0.6 (4)	C9—C10—O1—C12	−175.63 (18)

### Hydrogen-bond geometry (Å, º)

**Table:**

*D*—H···*A*	*D*—H	H···*A*	*D*···*A*	*D*—H···*A*
C5—H5*A*···O1^i^	0.96	2.57	3.316 (3)	135
C7—H7···N2^ii^	0.93	2.58	3.493 (2)	168

Symmetry codes: (i) −*x*+2, −*y*+1, −*z*+1; (ii) −*x*+1, −*y*+1, −*z*.

## References

[bb1] Altomare, A., Cascarano, G., Giacovazzo, C., Guagliardi, A., Burla, M. C., Polidori, G. & Camalli, M. (1994). *J. Appl. Cryst.* **27**, 435.

[bb2] Bernstein, J., Davis, R. E., Shimoni, L. & Chang, N.-L. (1995). *Angew. Chem. Int. Ed. Engl.* **34**, 1555–1573.

[bb3] Bruker (2004). *APEX2*, *SAINT* and *XPREP* Bruker AXS Inc., Madison, Wisconsin, USA.

[bb4] Farrugia, L. J. (2012). *J. Appl. Cryst.* **45**, 849–854.

[bb5] Gayathri, P., Jayabharathi, J., Srinivasan, N., Thiruvalluvar, A. & Butcher, R. J. (2010). *Acta Cryst.* E**66**, o1703. 10.1107/S1600536810022841 PMC3006898 21587923

[bb6] Rosepriya, S., Thiruvalluvar, A., Jayabharathi, J., Srinivasan, N., Butcher, R. J., Jasinski, J. P. & Golen, J. A. (2011). *Acta Cryst.* E**67**, o1065. 10.1107/S1600536811012098 PMC3089216 21754391

[bb7] Sheldrick, G. M. (2008). *Acta Cryst.* A**64**, 112–122. 10.1107/S0108767307043930 18156677

